# A new naphthalene-based fluorogenic substrate for cytochrome P450 4A11

**DOI:** 10.1042/BCJ20253130

**Published:** 2025-06-17

**Authors:** Dmitri R. Davydov, Kannapiran Ponraj, Nadezhda Davydova, Dilip Kumar Singh, Bhagwat Prasad

**Affiliations:** 1 Department of Chemistry, Washington State University, Pullman, WA 99164, U.S.A.; 2 Department of Pharmaceutical Sciences, Washington State University, Spokane, WA 99202, U.S.A.; † Division of Translational and Clinical Pharmacology, Cincinnati Children's Hospital Medical Center, Cincinnati, OH, 45229, USA

**Keywords:** 3-(6-methoxynaphthalen-2-yl)acrylic acid, CYP4A11, CYP1A2, fluorogenic substrates, high-throughput activity assay, human liver microsomes

## Abstract

We aimed to create a high-throughput fluorimetric assay for the activity of CYP4A11, the major 20-HETE-producing enzyme. To this end, we probed 3-(6-methoxynaphthalen-2-yl)acrylic acid (MONACRA) as a potential CYP4A11 substrate. We studied its metabolism using human liver microsomes (HLM) and recombinant P450 enzymes. O-demethylation of MONACRA by cytochromes P450 creates 3-(6-hydroxynaphthalen-2-yl)acrylic acid. The bright fluorescence of the product and its clear spectral resolution from the substrate allowed us to create a fluorimetric assay of MONACRA metabolism. We tested 16 recombinant human P450 enzymes and found noticeable demethylation activity only with CYP4A11 and CYP1A2. The *K_M_* for CYP4A11 is 189±37 μM, and the *k_cat_* accounts for 67±18 min^−1^. CYP1A2 exhibits a *K_M_* of 161±34 μM, with a *k_cat_* value of 44±6 min^−1^, although this enzyme also exhibited a decreased rate of turnover at high substrate concentrations, evidencing substrate inhibition with *K*_si_=650±200 μM. The studies with fluvoxamine and epalrestat, specific inhibitors of CYP1A2 and CYP4A11, respectively, showed that despite the activity of recombinant CYP1A2 with MONACRA, it does not take part in its metabolism in HLM. Thus, MONACRA can be utilized as a specific fluorogenic substrate of CYP4A11. We developed a robust and sensitive automated fluorimetric assay of MONACRA demethylation and used it to compare the substrate saturation profiles in seven pooled HLM preparations with the known composition of the P450 pool. These studies demonstrated a close correlation between the rate of the main kinetic phase of MONACRA metabolism and the fractional content of CYP4A11 in the P450 pool.

## Introduction

The membrane of the endoplasmic reticulum of liver cells is a home for two dozen cytochrome P450 species that function as an integrated multienzyme system [1-3]. The diversity of their substrate specificities and catalytic properties ensures the functional versatility of the P450 ensemble [4]. Besides its crucial role in drug metabolism, it also contributes to cellular signaling due to its role in synthesizing and disposing of a variety of secondary messengers and hormones [5,6]. Due to tight integration, any changes in the P450 composition under the influence of developmental, temporal, and environmental factors cause complex functional changes. They not only influence drug metabolism but also affect P450-dependent signaling pathways, such as those mediated by eicosanoids and retinoic acid. The interplay between xenobiotic metabolism and cytochrome P450-intermediated signaling plays an important role in health disorders [6]. In particular, they are central to such alcohol-induced pathologies as liver damage, carcinogenesis [7,8], and hypertension [9].

Studying the effects of alcohol consumption on the integral properties of the cytochrome P450 ensemble, we combine high-throughput metabolic assays with proteomic characterization of the P450 pool. We apply these techniques to human liver microsomes (HLMs) from donors with varying alcohol exposure to uncover its functional effects [10,11]. Application of this approach to explore the effects of alcohol consumption on P450-dependent cellular signaling requires assessing the activity of microsomal P450 species involved in the synthesis and disposal of signaling intermediates.

Key P450-dependent signaling pathways include those mediated by eicosanoids, the oxidized derivatives of 20-carbon polyunsaturated fatty acids, such as arachidonic acid (ARA) [12,13]. ARA is the substrate of several cytochrome P450 species catalyzing the formation of its bioactive epoxy, hydroxy, and dihydroxy derivatives. In particular, cytochrome P450 4A11 (CYP4A11) plays the prevailing role in the synthesis of 20-hydroxyeicosatetraenoic acid (20-HETE), one of the most critical bioactive products of P450-dependent oxidation of ARA [14,15]. 20-HETE has a wide range of effects on the vascular system and regulates renal function and blood pressure [16,17]. In the liver, it exerts essential functions in lipid homeostasis and controls fat-dependent energy supply and metabolism. It also acts as an inflammatory mediator and is important in inflammatory diseases [18]. In this view, studying the impact of the changes in the composition of the P450 ensemble on the activity of CYP4A11 is vital for understanding its role in the mechanisms of health disorders caused by environmental factors and alcohol-induced hypertension, inflammation, carcinogenesis, and liver damage in particular.

A critical impediment to establishing a high-throughput method for studying functional properties of CYP4A11 in HLM is a lack of a simple and cost-effective tool for monitoring its activity suitable for high-throughput setups. The most common method for assessing the enzyme activity is based on determining the products of the metabolism of lauric acid by LC-MS [19]. However, this method lacks specificity to CYP4A11, as several other P450 species also metabolize this substrate. The only known specific approach for measuring CYP4A11 activity is the luminogenic assay with a CYP4A11-specific substrate – the derivative of luciferin, luciferin 4A [20-22]. However, a high-throughput implementation of luminogenic assays is unwieldy, expensive, and requires special equipment. Furthermore, the production of Luciferin-4A by Promega Corporation is now discontinued, and it is no longer commercially available.

Fluorogenic substrates offer the most cost-effective alternatives to LC-MS or luminogenic assays for high-throughput use. However, unlike most microsomal P450 drug-metabolizing enzymes [23-26], CYP4A11 has no known specific fluorogenic substrate that could be used in the assays of its activity in HLM.

We present the design and characterization of the first CYP4A11-specific fluorogenic substrate, 3-(6-methoxynaphthalen-2-yl)acrylic acid (MONACRA). Probing 16 recombinant human P450 enzymes with this substrate, we demonstrated that only CYP4A11 and CYP1A2 metabolize MONACRA at a noticeable rate. Yet, inhibitory analysis with specific inhibitors of CYP4A11 and CYP1A2 demonstrated that CYP1A2 is not involved in MONACRA metabolism in HLM. Thus, MONACRA can be used as a specific fluorogenic substrate for CYP4A11. Based on these findings, we developed a robust and sensitive automated fluorimetric assay for MONACRA demethylation applicable to high-throughput screening of CYP4A11 activity in a large series of HLM preparations.

## Results and discussion

### Identification of MONACRA as a potential fluorogenic substrate of CYP4A11

Most known fluorogenic substrates of cytochromes P450 are the derivatives of coumarin and resorufin [24-26]. In most cases, the fluorimetric detection is based on a prominent increase and red shift of fluorescence of these compounds after P450-dependent removal of substituents in their methoxy, ethoxy, or benzyloxy groups. The naphthalene moiety can also be used to construct fluorogenic P450 substrates based on the same principle – incorporation of a hydroxy group or dealkylation of an alkoxy substituent in the naphthalene ring also results in an increase and red shift of fluorescence. So far, the only naphthalene-based fluorogenic substrate used in P450 research is 2-naphthoic acid. This compound is a fluorogenic substitute for cinnamic acid, a substrate of plant cytochromes P450 involved in lignin biosynthesis [27,28]. In this case, P450-dependent hydroxylation forms 6-hydroxy-2-naphthoic acid, possessing ample fluorescence at 450 nm with an excitation maximum positioned at 295 nm.

P450-dependent O-demethylation of methoxy-substituted naphthalenes can form the same kind of fluorescent products. An example of a known P450 substrate of this kind is naproxen, 6-methoxy-α-methyl-2-naphthaleneacetic acid, a nonsteroidal anti-inflammatory drug that undergoes CYP1A2- and CYP2C9-catalyzed O-demethylation in human liver [29]. However, despite the contrasting difference between naproxen and desmethylnaproxen in fluorescent properties [30], fluorimetric detection has never been employed in studying naproxen metabolism.

**Figure 1:**
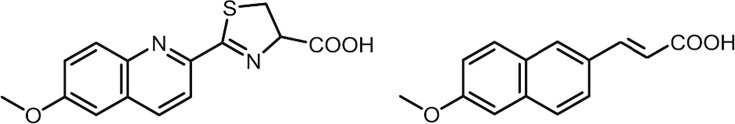
Structures of luciferin 4A (left) and 3-(6-methoxynaphthalen-2-yl)acrylic acid (MONACRA, right).

In developing a fluorogenic substrate specific for CYP4A11, we relied on the structure of luciferin 4A, the only known luminogenic substrate of CYP4A11 [20]. As seen in Figure 1, the luciferin 4A structure involves a methoxyquinidine moiety. We hypothesized that it could be replaced with a closely similar methoxynaphthalene without losing the substrate affinity to CYP4A11.

**Figure 2:**
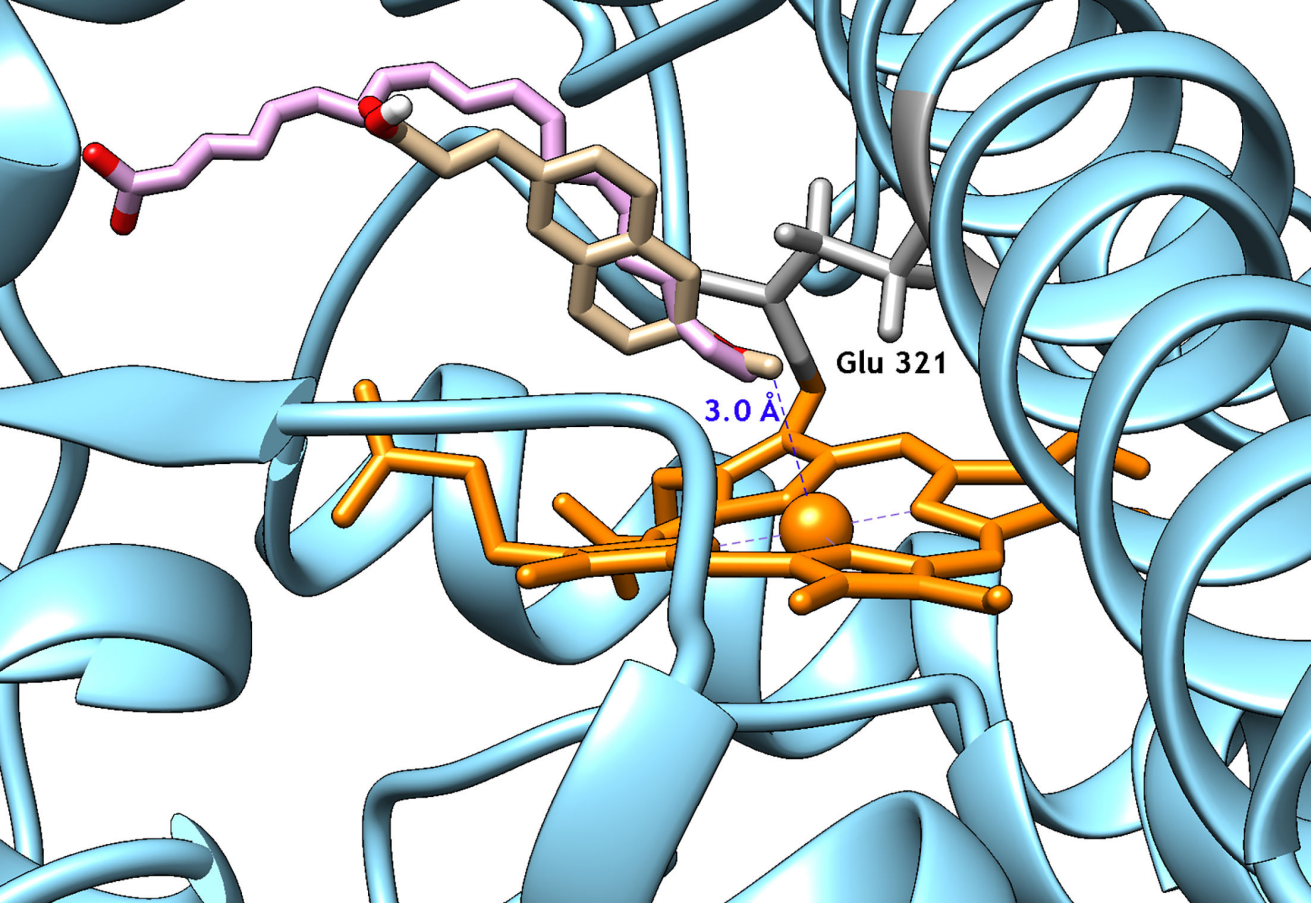
Docking of arachidonic acid (purple) and 3-(6-methoxynaphthalen-2-yl)acrylic acid (MONACRA; tan) molecules into the substrate binding pocket of CYP4A11. The structural model of CYP4A11 was generated by AlphaFold and the porphyrin moiety (orange) covalently connected to the Glu_321_ side chain (gray) was inserted by superimposing the AlphaFold-generated structure of the protein chain with the crystal structure of CYP4B1 (PDB entry 5T6Q). This image was produced using the Chimera 1.18 package from the Computer Graphics Laboratory, University of California, San Francisco (http://www.cgl.ucsf.edu/chimera/, [33])

Thus, we decided to probe the derivatives of methoxynaphthalene as CYP4A11 substrates. The simplest potential methoxynaphthalene-based P450 substrate is 6-methoxy-2-naphthoic acid, a close analog of naproxen, which is known to be metabolized by P450 enzymes [29]. We probed this substrate as a P450 substrate and found that it undergoes O-demethylation catalyzed predominantly by CYP1A2. However, it is not metabolized by CYP4A11 (data not shown). This molecule is apparently too small to be a CYP4A11 substrate. Thus, we searched commercially available derivatives of methoxynaphthalene to find a compound with a longer carboxylic acid chain substituent in the naphthalene ring. This search identified MONACRA (Figure 1) as a potential CYP4A11 substrate. Using Autodock Vina [31] to dock this molecule into a model of CYP4A11 built with AlphaFold [32], we found that it fits into the substrate binding pocket with calculated ΔG° of interactions of −9.1 kcal/mol, which is even more supportive of binding than that calculated for the CYP4A11 complex with ARA (−8.0 kcal/mol). As illustrated in Figure 2, the predicted position of MONACRA in the binding pocket is similar to that of the ARA and compatible with its CYP4A11-catalyzed O-demethylation. Inspired by these results, we probed MONACRA as a substrate for cytochromes P450 in the human liver. We attempted to design a fluorimetric assay of its metabolism suitable for high-throughput studies in HLM.

### Probing MONACRA as a substrate of microsomal P450 enzymes

#### Detection of MONACRA demethylation by fluorimetric assay of formed formaldehyde.

To probe if MONACRA can be metabolized to hydroxy-naphthyl acrylic acid (HONACRA) by liver microsomes, we utilized our high-throughput fluorimetric assay of formed formaldehyde with acetoacetanylide (FA-AAA assay, [34]). In our initial experiments, we used cynomolgus monkey (*Macaca fascicularis*) liver microsomes (MLM). Cynomolgus monkey liver microsomes possess the complete set of homologs of human CYP4 enzymes active in ARA metabolism [35], and the degree of sequence identity of monkey CYP4A11 (NCBI accession number AAZ29443.1) with its human homolog (AAB29502.1) is as high as 95.4%.

**Figure 3:**
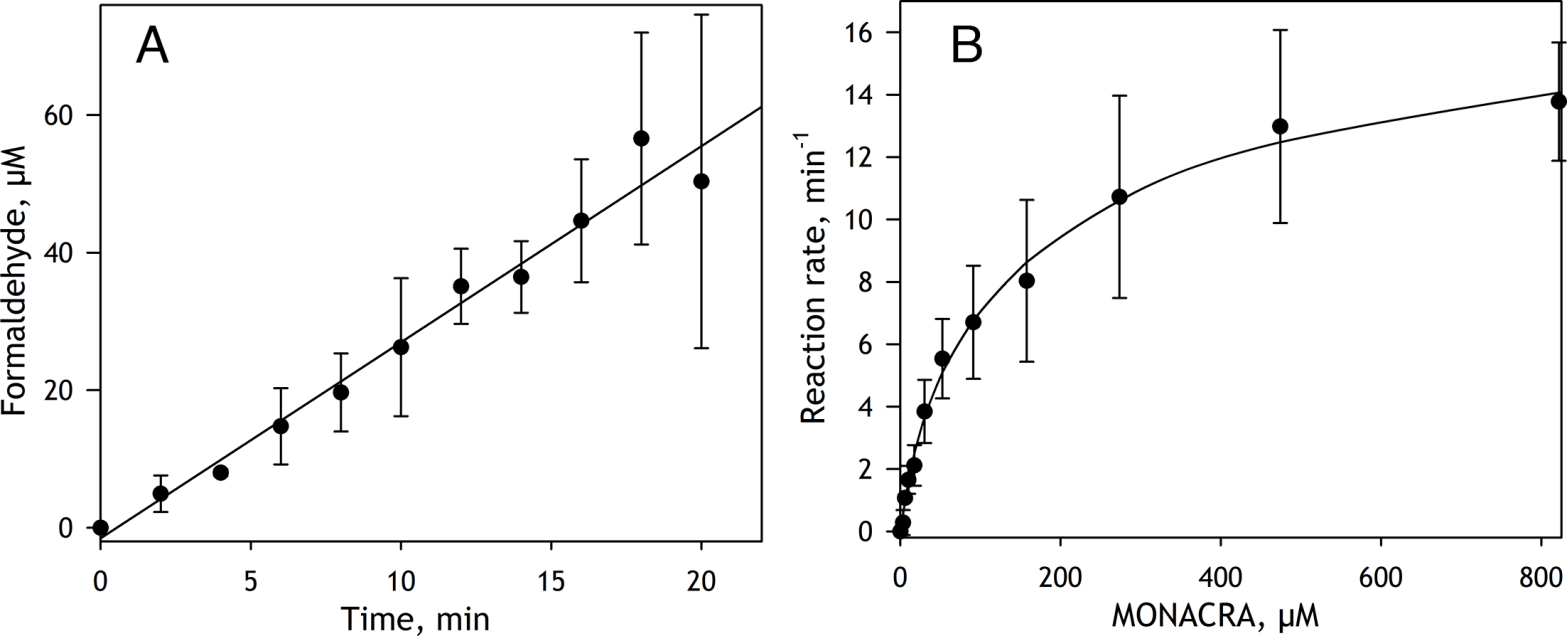
O-demethylation of 3-(6-methoxynaphthalen-2-yl)acrylic acid (MONACRA) by monkey liver microsomes (MLM). Panel **A** shows a kinetic trace of the accumulation of formaldehyde formed upon incubation of 2.5 mg/ml MLM (~0.3 µM of microsomal CPR) with 300 µM MONACRA in the presence of NADPH-generating system. Panel **B** exemplifies the substrate saturation profile of MONACRA demethylation by MLM (0.98 mg/ml) determined by formed formaldehyde with acetoacetanylide (FA-AAA) assay with 20-min incubation at 30°C. The datasets shown in the figures represent averages of the results of two individual experiments, and the error bars indicate the standard deviations.

As shown in Figure 3A, incubation of MLM with 400 µM MONACRA in the presence of the NADPH-generating system results in the production of formaldehyde, which remains linear for at least 20 min. The dependence of the reaction rate on MONACRA concentration obtained with MLM and Formaldehyde (FA) assay is shown in Figure 3B. Using the non-linear regression algorithms implemented in SpectraLab software (see Materials and Methods), we were able to adequately approximate this substrate saturation profile (SSP) with a combination of two Michaelis–Menten equations with *K*_M_ values of 28.4 and 260 µM and the total *V*_max_ of 17.2 mol FA per mol of NADPH-cytochrome P450 reductase (CPR) per minute.

#### Exploring the process of MONACRA demethylation by LC-MS

**Figure 4:**
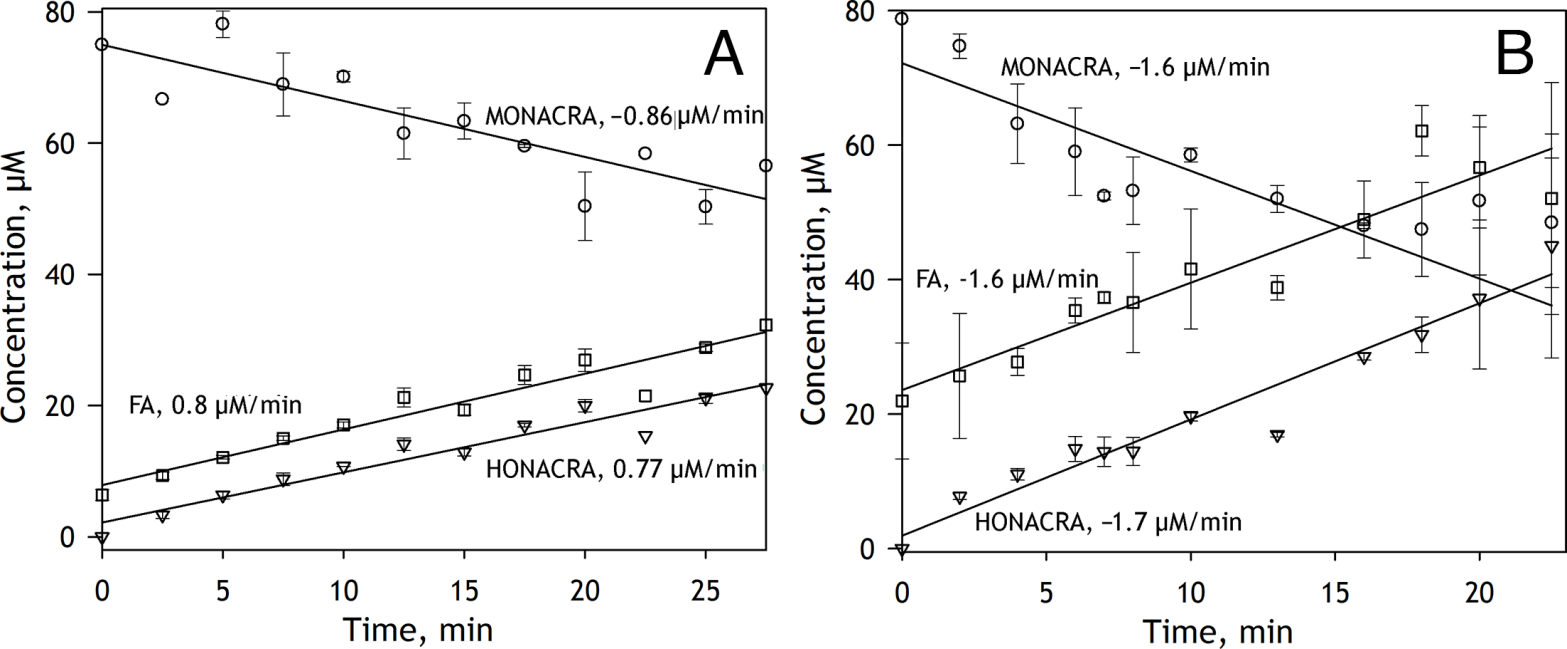
Kinetics of O-demethylation of 3-(6-methoxynaphthalen-2-yl)acrylic acid (MONACRA) studied by LC-MS. Panel **A** shows the plots of time-dependent changes of MONACRA (circles), hydroxy-naphthyl acrylic acid (HONACRA; triangles), and formaldehyde-derived 3,5-di-N-phenylacetyl-1,4-dihydrolutidine (DPDL) (squares) upon incubation of 0.125 µM CYP1A2 Supersomes with 75 µM MONACRA and NADPH-generating system at 36°C. The datasets represent the averages of the results of two individual experiments, and the error bars indicate the standard deviations. Panel **B** exemplifies the same plots obtained in the experiment with 0.075 µM CYP4A11 Supersomes and 78.8 µM MONACRA. The datasets for MONACRA and HONACRA shown in this figure are the averages of the results of two independent experiments. The formaldehyde accumulation dataset represents an average of the results of LC-MS and fluorimetric determinations of DPDL in the same experiment.

Probing the activity of individual recombinant cytochrome P450 enzymes in Supersomes® in catalyzing O-demethylation of MONACRA, we identified CYP1A2 and CYP4A11 as the only human enzymes capable of MONACRA metabolism (vide infra for more details). We then studied the time courses of MONACRA depletion and formation of formaldehyde and HONACRA by these enzymes with LC-MS/MS. In these experiments, we used the conversion of formaldehyde to 3,5-di-N-phenylacetyl-1,4-dihydrolutidine (DPDL) via Hantzsch reaction with acetoacetanilide [34]. The results of these experiments are illustrated in Figure 4.

As seen from this figure, the metabolism of MONACRA by CYP4A11 and CYP1A2 results in the accumulation of the products of its O-demethylation, formaldehyde, and HONACRA, the rates of which are commensurate with the rate of substrate depletion. These results point out O-demethylation as the only pathway for MONACRA metabolism by both CYP4A11 and CYP1A2.

#### Elaborating the fluorimetric assay of MONACRA demethylation

MONACRA fluorescence excitation spectrum has a main maximum positioned at 343 nm and a prominent shoulder centered at 298 nm. The spectrum of MONACRA emission represents a broad band spanning from 380 to 550 nm with a maximum at 440 nm. Since the product of MONACRA demethylation (HONACRA) is not commercially available and its fluorescent properties are not known, we had to reconstruct its emission and excitation spectra based on monitoring the kinetics of MONACRA demethylation with scanning fluorimetry.

**Figure 5:**
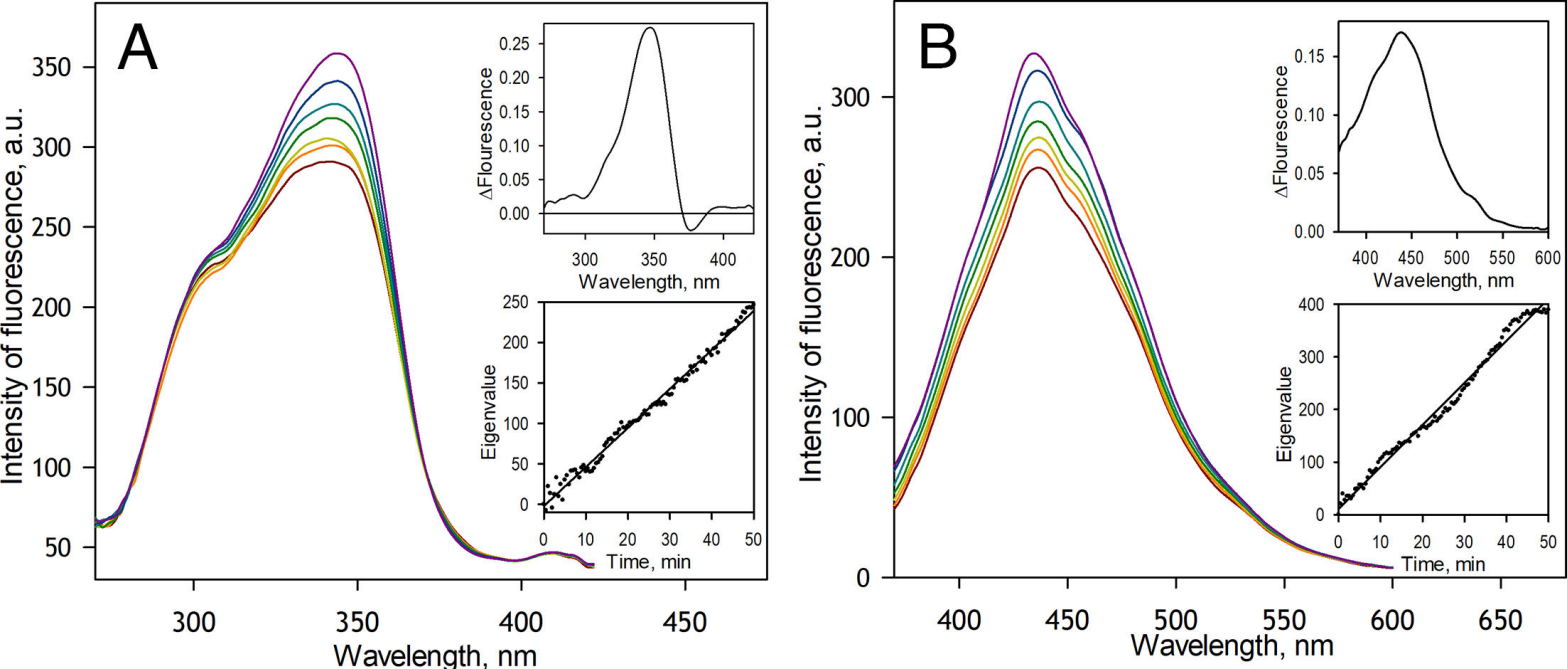
Changes in the spectra of excitation (**A**) and emission (**B**) of 3-(6-methoxynaphthalen-2-yl)acrylic acid (MONACRA) fluorescence caused by its O-demethylation. The spectra were recorded in 30-sec intervals during incubation of 1.7 mg/ml MLM with 200 µM MONACRA in 0.1 M Na-Hepes buffer, pH 7.4, containing 60 mM KCl and NADPH-generating system at 36°C. The main panels of the graphs exemplify the spectra recorded after 0 (brown), 10, 20, 30, 40, and 50 (purple) min of incubation. The insets show the results of applying principal component analysis (PCA) to these spectral series. The top insets show the spectra of the first principal component (>99.5% of the overall spectral changes), whereas the time dependencies of the respective eigenvalues are shown in the bottom insets. Spectra of excitation were recorded at the emission wavelength of 440 nm, whereas the emission spectra correspond to excitation at 350 nm. The slits of the excitation and emission monochromators were set to 5 nm bandwidth, and the voltage at the PMT was set to 600 V.

**Figure 6:**
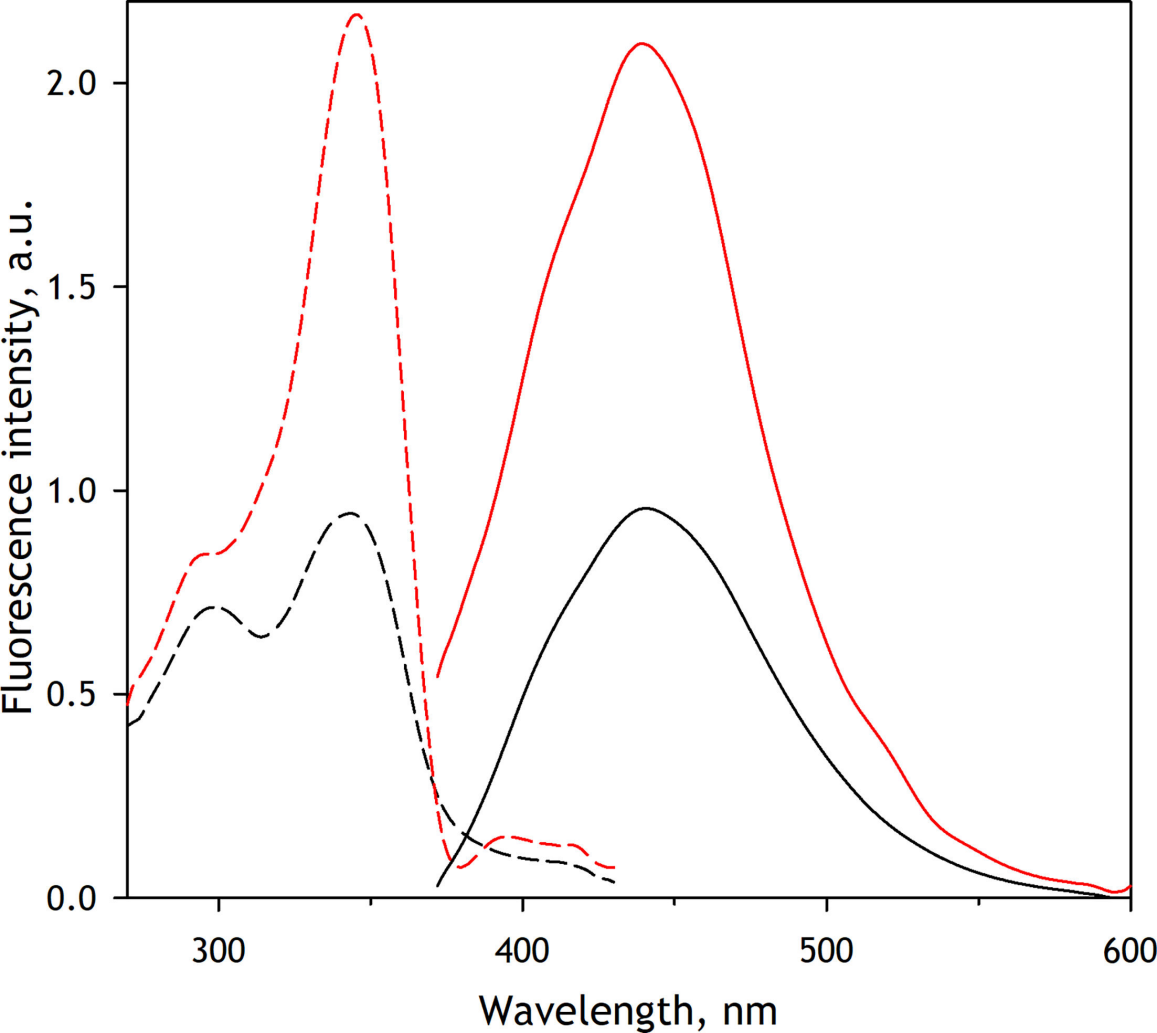
Spectra of excitation (dashed lines) and emission (solid lines) of 3-(6-methoxynaphthalen-2-yl)acrylic acid (MONACRA; black) and hydroxy-naphthyl acrylic acid (HONACRA; red). The spectra correspond to the fluorescence of 1 µM substances in Hepes buffer, pH 7.4, measured with a Cary Eclipse spectrofluorometer in a 5 × 5 quartz cell at PMT voltage of 600 V and excitation and emission slits set to 5 nm bandwidth. The spectra of excitation were recorded at the emission wavelength of 440 nm. The spectra of emission correspond to excitation at 350 nm.

The series of excitation and emission spectra recorded during the incubation of MLM with MONACRA in the presence of the NADPH-generating system is shown in Figure 5. As seen from these graphs, O-demethylation of MONACRA increases the intensity of fluorescence while having virtually no effect on the shape of the spectra. Quantifying the formation of formaldehyde in these experiments with our FA-AAA fluorimetric assay allowed scaling the observed spectral changes to 1 µM of formed formaldehyde and, thus, reconstituting the spectra of HONACRA fluorescence. The spectra of excitation and emission of HONACRA reconstituted in this way are shown in Figure 6, along with the spectra of MONACRA fluorescence.

As demonstrated by these results, O-demethylation of MONACRA results in an approximately two-fold increase in its fluorescence intensity. Real-time monitoring of MONACRA demethylation can be performed by following the increase in fluorescence at 440 nm with excitation at 345 nm. However, the sensitivity of this assay is low. Furthermore, it is not applicable for high-throughput acquisition of substrate-saturating profiles that requires resolving the fluorescence of the product from that of the substrate. In this view, we tried to improve the method of fluorimetric monitoring of MONACRA demethylation by alkalizing the assay medium. The differences in the intensities and the positions of maxima of fluorescence between methoxy-derivatives of naphthalene compounds with acidic substituent groups and their demethylated derivatives may escalate at alkaline pH, as it was evidenced by Konstantinos and co-authors in the case of naproxen [30].

**Figure 7:**
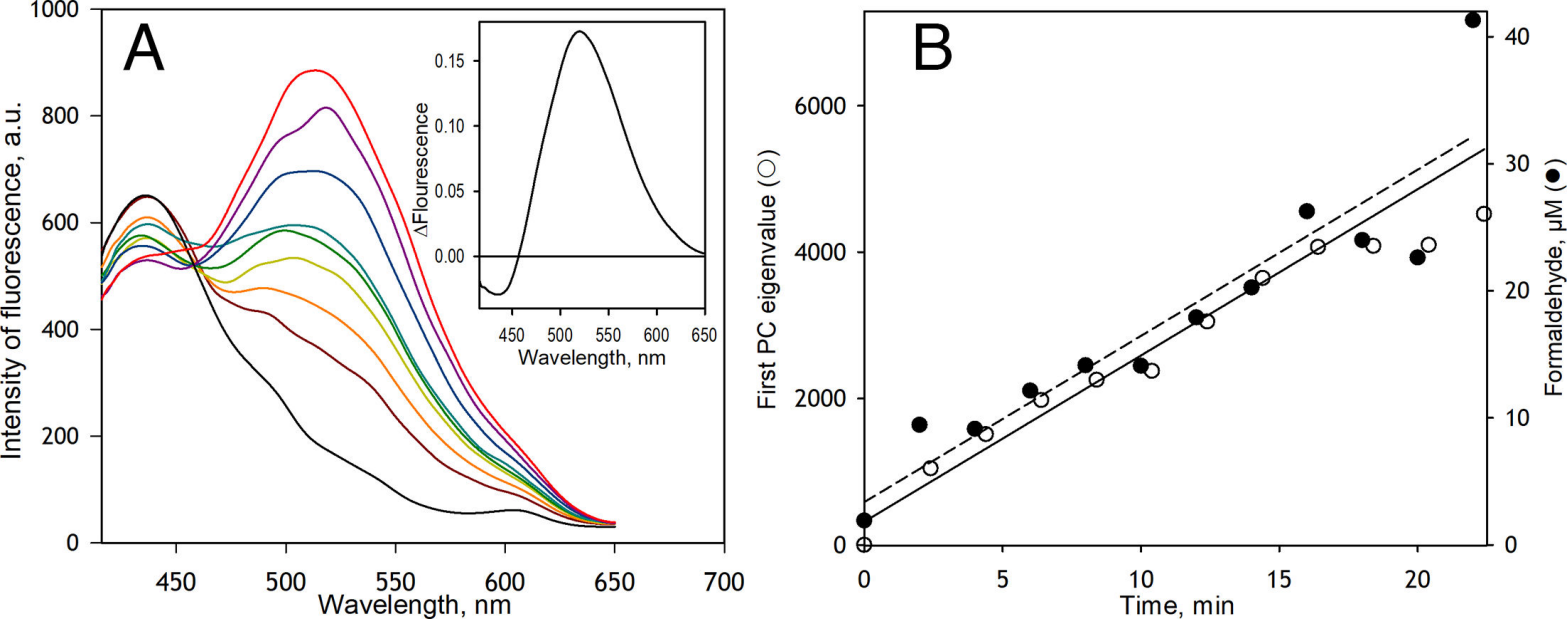
The time course of 3-(6-methoxynaphthalen-2-yl)acrylic acid (MONACRA) demethylation by monkey liver microsomes (MLM) monitored by spectra of fluorescence at pH 10.4. The emission spectra (excitation at 375 nm) shown in panel **A** represent the samples quenched in 2-min intervals after starting the reaction by adding MLM, where the spectra shown in black and red correspond to the time points of 0 and 16 min, respectively. The reaction mixture containing 400 µM MONACRA, 60 mM KCl, 1.7 mg/ml MLM, and NADPH-generating system in 0.1 M Na-Hepes buffer, pH 7.4, was incubated at 36° in a multiwell plate placed in a shaking incubator. The inset in panel **A** shows the spectrum of the first principal component (99.3% of the overall spectral changes) obtained by applying PCA to this spectral series. Panel **B** shows the time dependence of the respective eigenvalues (open circles) along with the time course of the increase in formaldehyde concentration determined by FA-AAA assay (closed circles).

To explore this possibility, we developed a multiwell-plate-based assay, where the reaction mixture incubated in the wells is first quenched by trichloroacetic acid. Then, we alkalized it by adding concentrated Na-glycine buffer with pH 10.4 (see Materials and Methods). A series of spectra of fluorescence emission recorded during the incubation of MLM with 400 µM MONACRA obtained in this way are shown in Figure 7. As seen from Figure 7A, when the emission spectra are recorded at alkaline pH, they exhibit a spectacular fluorescence increase with the emission maximum centered around 510 nm. This increase goes side by side with an increase in the concentration of FA, the product of MONACRA demethylation (Figure 7B).

**Figure 8:**
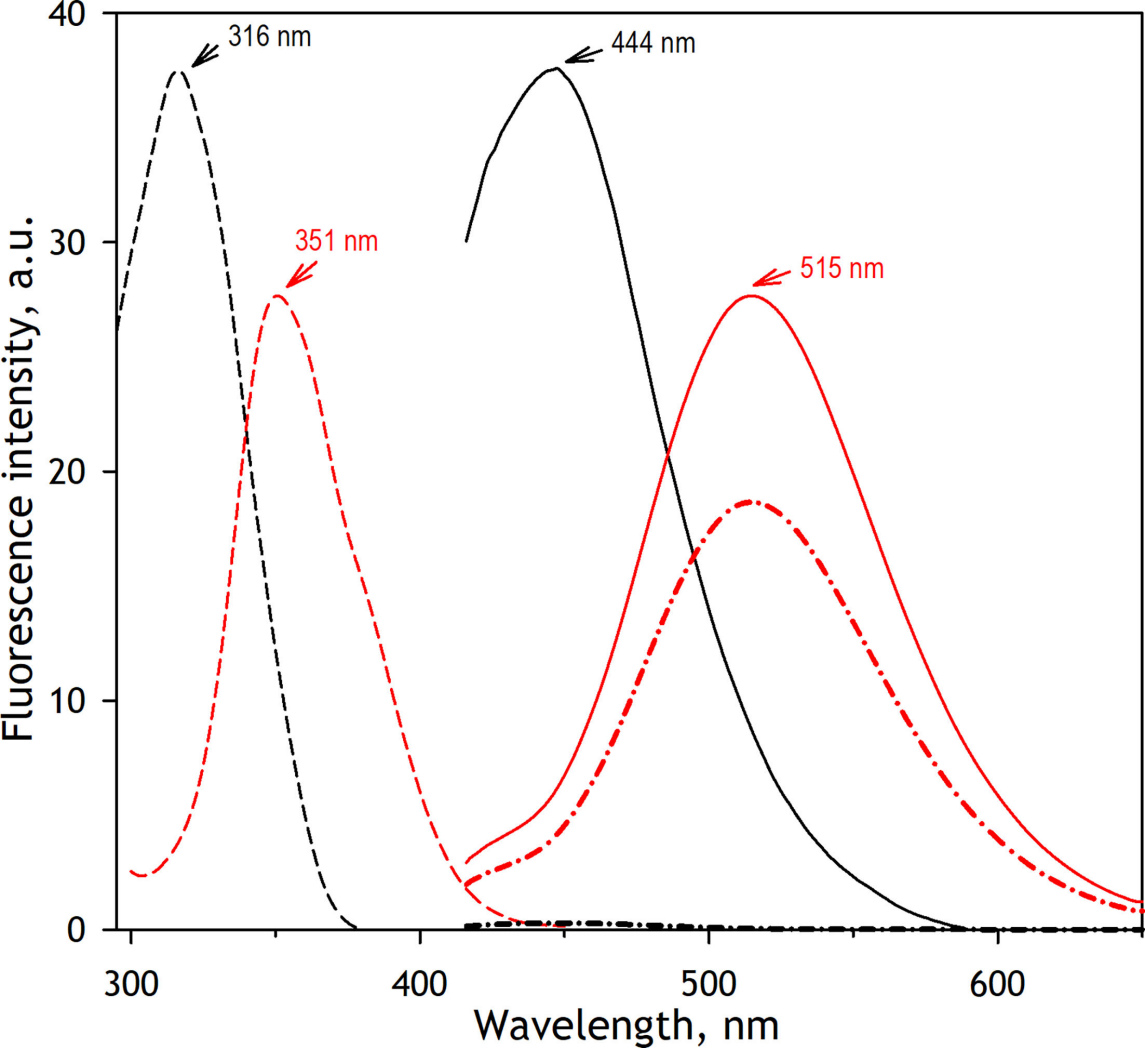
Spectra of fluorescence of 3-(6-methoxynaphthalen-2-yl)acrylic acid (MONACRA; black) and hydroxy-naphthyl acrylic acid (HONACRA; red) at pH 10.4. The plots in thin dashed and solid lines represent the spectra of excitation and emission, respectively, whose amplitudes correspond to emission and excitation at the respective maximum. The plots shown in bold dashed-and-dotted lines correspond to the spectra of emission of MONACRA and HONACRA recorded with excitation at 375 nm.

We reconstituted the fluorescence spectra of HONACRA at pH 10.4 using an approach analogous to that used for obtaining the fluorescence spectra at neutral pH described above. However, in this case, we didn’t rely on the time dependence of fluorescence. Instead, we used the series of spectra from experiments that tracked spectral changes at different MONACRA concentrations. Combining these experimental results with formaldehyde quantification, let us recreate the fluorescence spectra. This strategy is illustrated by Figure S1 in the Supplementary Material. The spectra of fluorescence of HONACRA obtained in this way, along with the spectra of MONACRA fluorescence, are shown in Figure 8.

As seen in Figure 8, alkalization of the assay medium results in a perfect resolution of the excitation and emission bands of the product from those of the substrate. When the fluorescence is excited at 375 nm (bold dashed-and-dotted lines in Figure 8), the fluorescence of MONACRA is negligible. In contrast, the intensity of HONACRA fluorescence is as high as two-thirds of its level observed with excitation at the maximum (351 nm).

Based on these observations, we developed a high-throughput automated assay applicable for studying SSPs of MONACRA metabolism by microsomal P450 enzymes. In this procedure, the incubation mixtures containing an NADPH-generating system and various substrate concentrations are placed in a multiwell plate and preincubated at 30°C in a thermostated shaker. The reaction is started by adding microsomal suspension to the final concentration of 0.3–1 mg/ml of microsomal protein or 0.02–0.1 µM of recombinant P450 enzymes in Supersomes. Following the incubation for 20 min at 30°C, the reaction is quenched by adding trichloroacetic acid (final concentration 86 µM). After supplementing the wells with Na-glycine buffer (pH 10.4, final concentration 2.1 M), the plates are subjected to scanning the fluorescence spectra with excitation at 375 nm. This assay, which is described in detail in Materials and Methods, allows reliable detection of the concentrations of HONACRA as low as 0.1 µM.

Due to the unavailability of the product of MONACRA demethylation (HONACRA), the calibration of the assay may require measuring the amount of produced formaldehyde and involve the application of principal component analysis (PCA) to data analysis. In order to simplify the calibration procedure, we introduced Coumarin 152 (C152) as a HONACRA substitute. As shown in Figure S2 in Supplementary Material, the fluorescence spectrum of this laser dye is closely similar to that of HONACRA. According to our measurements, the fluorescence intensity of C152 in our assay media at pH 10.4 with excitation at 375 nm is 2.1-fold lower than that of HONACRA. Thus, the spectrum of fluorescence of 2.1 µM C152 may be used as a substitute for the spectrum of fluorescence of 1 µM MONACRA for calibration purposes.

#### Metabolism of MONACRA by individual recombinant P450 enzymes

We used the high-throughput activity assay described above to probe MONACRA metabolism by 16 human microsomal P450 enzymes, namely CYP1A2, CYP2A6, CYP2B6, CYP2C8, CYP2C9, CYP2C19, CYP2D6, CYP2E1, CYP2J2, CYP3A4, CYP3A5, CYP4A11, CYP4F2, CYP4F3A, CYP4F3B, and CYP4F12. In these experiments, we used human P450 enzymes co-expressed with NADPH-cytochrome P450 reductase (CPR) in microsomes of insect cells transfected by recombinant baculovirus (Supersomes). All those preparations, except for the CYP1A2 and CYP4A11 Supersomes, also contained human cytochrome *b*_5_ co-expressed.

**Figure 9:**
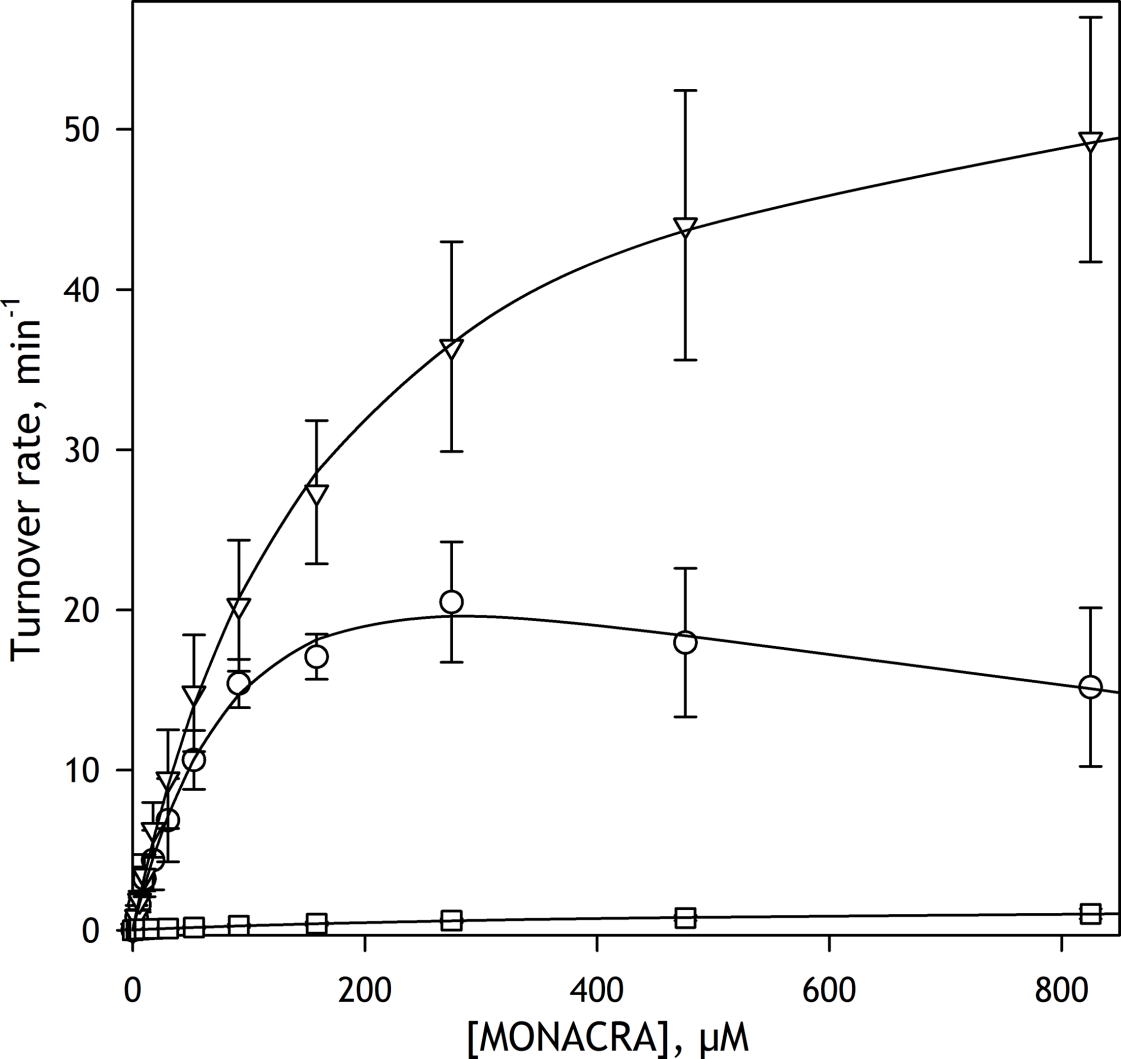
Substrate saturation profiles (SSPs) of 3-(6-methoxynaphthalen-2-yl)acrylic acid (MONACRA) metabolism by CYP4A11 (triangles), CYP1A2 (circles), and CYP2C8 (squares) in Supersomes. The reaction rate is expressed as mol of product per mol of P450 per minute (min^−1^). The datasets shown in the graph represent the averages of the results of 5 (CYP4A11), 6 (CYP1A2) or 4 (CYP2C8) individual experiments. The solid lines represent the results of fitting the datasets to the Michaelis–Menten equation (CYP4A11 and CYP2C8) or the equation of substrate inhibition (CYP1A2).

Among all probed P450 enzymes, detectable activity in MONACRA demethylation was observed only with CYP4A11, CYP1A2, and CYP2C8. The SSPs of MONACRA demethylation by these enzymes are exemplified in Figure 9. As seen from this figure, the highest turnover rate was observed with CYP4A11. Although the rate of metabolism of MONACRA by CYP1A2 at low substrate concentrations was comparable to that of CYP4A11, CYP1A2 exhibited pronounced substrate inhibition at high substrate concentrations. The rate of MONACRA metabolism by CYP2C8 is negligibly small. The kinetic parameters of MONACRA metabolism by these three enzymes are shown in Table 1.

**Table 1:** Parameters of 3-(6-methoxynaphthalen-2-yl)acrylic acid (MONACRA) demethylation by CYP4A1, CYP1A2, and CYP2C8 in Supersomes.^1^

Enzyme	*K_M_*, µM	*K_si_*, µM^2^	*V_max_*, min^−1^
CYP4A11	189±37		66.8±17.5
CYP1A2	161±34	648±197	43.7±5.9
CYP2C8	456±188		1.6±0.7

1 The values in the table represent the averages of the results of fitting 4–6 individual datasets to the Michaelis–Menten (CYP4A11 and CYP2C8) or substrate inhibition (CYP1A2) equation. The ± values are the confidence intervals calculated for *P*=0.05.

2 Value of the substrate inhibition constant derived from fitting CYP1A2 data to the substrate inhibition equation (eqn 2).

### Probing the role of individual P450 enzymes in MONACRA metabolism by HLM

#### Metabolism of MONACRA by pooled HLM preparations

**Figure 10:**
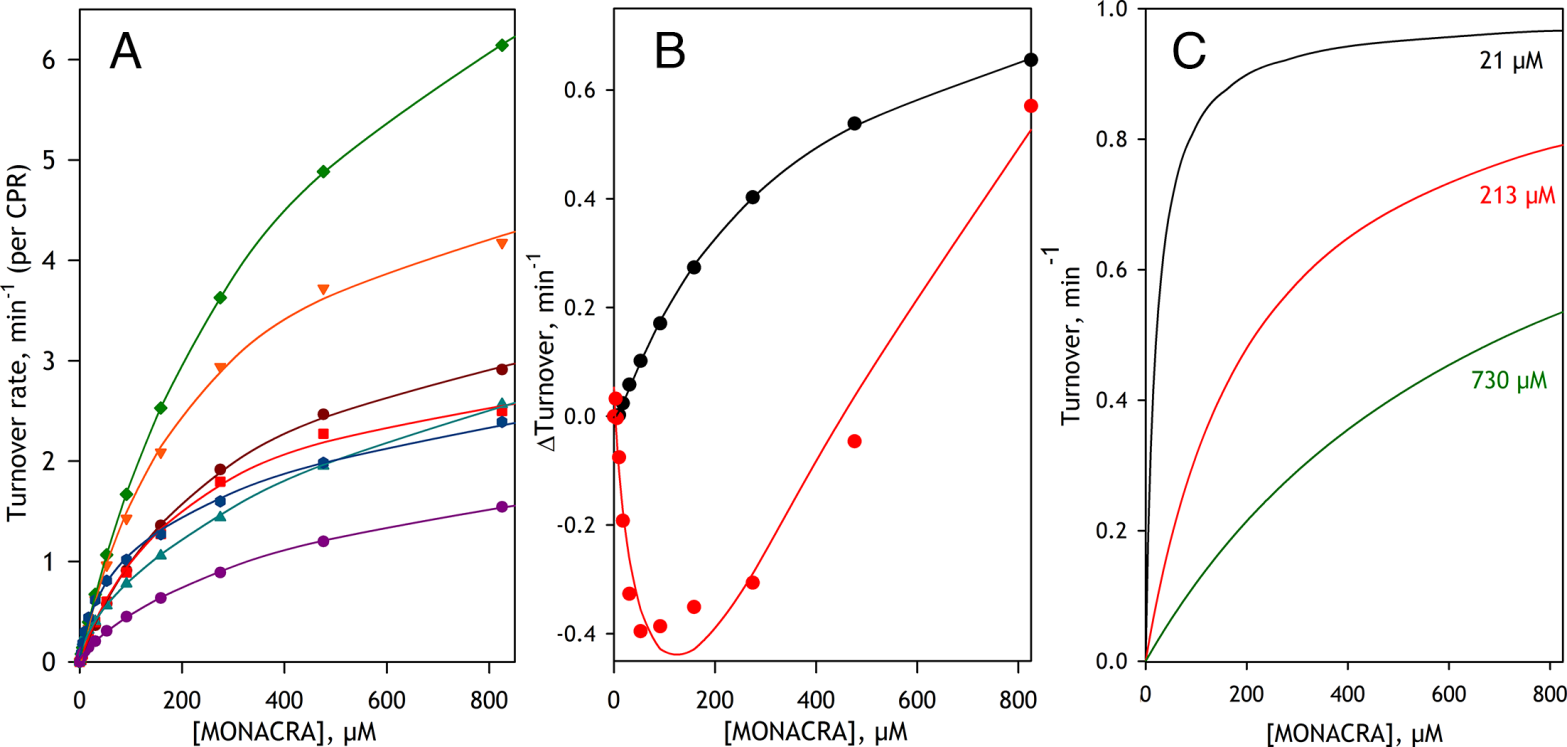
Substrate saturation profiles (SSPs) of 3-(6-methoxynaphthalen-2-yl)acrylic acid (MONACRA) metabolism by seven pooled preparations of human liver microsomes (HLMs) and their global analysis. The HLM preparations used in this study and their designations are described in the “Materials and Methods” section of the article. Panel (**A**) shows the SSPs obtained with HLM preparations CDN (brown), DNJ (orange), EGW (red), WGP (green), X096 (light blue), X263 (dark blue), and X347 (purple) along with their approximations (solid lines) obtained from global fitting of the dataset with a combination of three Michaelis–Menten equations. Panel (**B**) shows the plots of the first (black) and the second (red) principal components found by PCA applied to the shown dataset. These two principal components cover 99.4% of the overall differences between the SSPs. Panel (**C**) shows three Michaelis–Menten components obtained from the global fitting of the dataset and used to approximate the traces shown in panels **A** and **B**.

To probe the MONACRA metabolism by the human liver P450 system, we explored the SSPs of our new fluorogenic substrate by seven different pooled HLM preparations with P450 pool composition characterized by a global proteomics toolkit [11,36]. The set of SSPs derived from this study is shown in Figure 10A. As seen from this graph, the studied preparations exhibit a significant difference in the amplitude and the shape of SSPs. The results of applying PCA to this combined dataset are shown in Figure 10B. The first two principal components cover 99.4% of the overall differences between the individual SSPs. While the first principal component mainly reflects the differences in SSP amplitude, the second PC is responsible for the changes in the contribution of the high- and low-affinity components. Global fitting of the dataset with a combination of three Michaelis–Menten equations yielded the components with *K*_M_ values of 21, 213, and 730 µM (Figure 10C). The combinations of these three Michaelis–Menten components approximate all individual SSPs of the dataset with a square correlation coefficient (*R* ^2^) higher than 0.998. According to this approximation, the fraction of the high-affinity phase varies from 0 to 21% (average of 5.5%). The middle-affinity phase is responsible for 0–100% of metabolism (average of 49.2%), and the lowest affinity component accounts for 0–90% of the total amplitude with an average of 45.3%.

**Figure 11:**
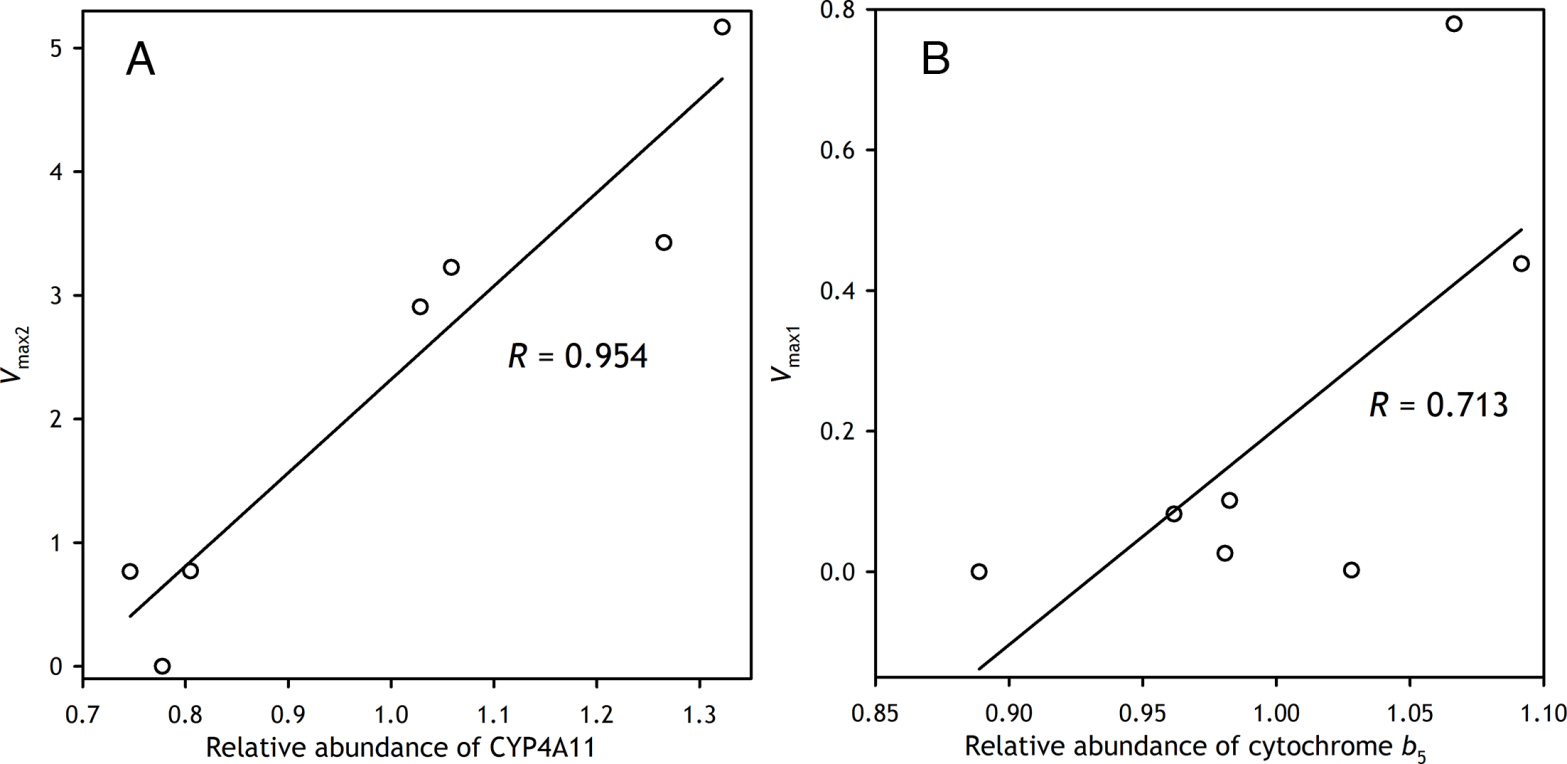
Correlations between the amplitude of the middle-affinity phase and relative abundance of CYP4A11 (**A**) and between the amplitude of the high-affinity phase and relative abundance of cytochrome *b*_5_ (**B**).

We analyzed the relationship between the amplitudes of individual Michaelis–Menten components and the P450 pool composition in pooled HLM preparations (refer to [36] for the dataset). Our results showed a strong correlation (*R*=0.954) between the amplitude of the middle-affinity component (*K*_M_=213 µM) and the relative abundance of CYP4A11 (see Figure 11A). In contrast, we did not find significant correlations between the high- and low-affinity phase amplitudes and the levels of 11 probed P450 species (CYP1A2, CYP2A6, CYP2B6, CYP2C8, CYP2C9, CYP2C19, CYP2D6, CYP2E1, CYP3A4, CYP3A5, and CYP4A11). However, the amplitude of the high-affinity component, which ranges from 0 to 21% of the total SSP amplitude, reveals a modest correlation with the abundance of cytochrome *b*_5_ (*b*_5,_ *R*=0.713, Figure 11B), suggesting a role of this protein in modulating MONACRA metabolism. It is to be noted that the preparations of Supersomes used in our initial experiments did not contain cytochrome *b*_5_ co-expressed. It is plausible, therefore, that *b*_5_-containing Supersomes, if available, would exhibit a higher affinity of CYP4A11 to MONACRA due to the potential effect of *b*_5_ on CYP4A11 interactions with the substrate.

#### Probing the role of CYP4A11 and CYP1A2 in MONACRA metabolism by inhibitory analysis

**Figure 12:**
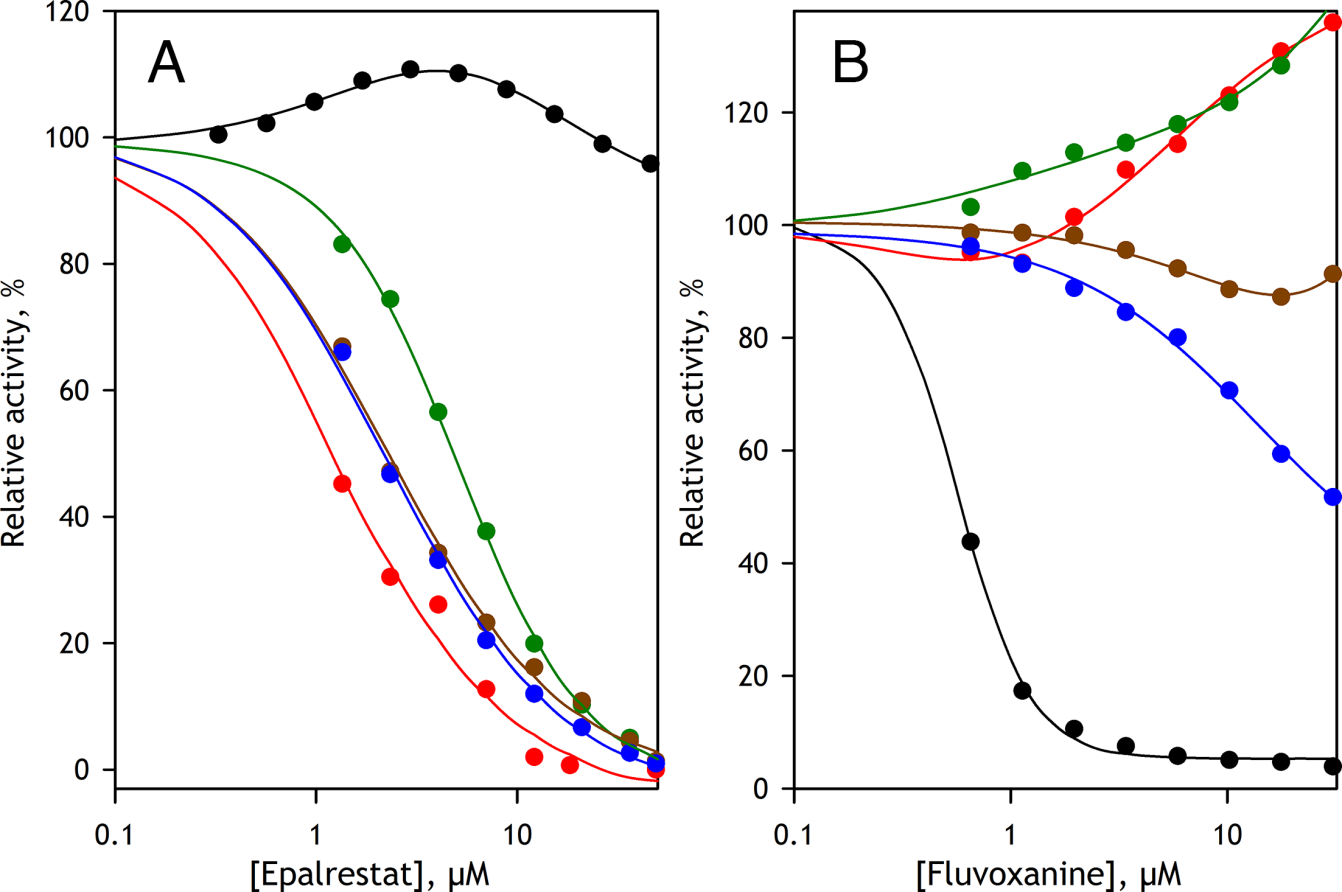
Effect of epalrestat (**A**) and fluvoxamine (**B**) on 3-(6-methoxynaphthalen-2-yl)acrylic acid (MONACRA) demethylation by recombinant CYP1A2 (black) and CYP4A11 (red) and three pooled human liver microsome (HLM) preparations: X263 (green), DNJ (blue), and WGP (brown). Solid lines represent the approximation of the datasets by eqn 1 (in the instances of pure inhibition) or a combination of two hyperbolic equations (in the cases of activation or combined inhibition and activation). All experiments were carried out at 150 µM MONACRA concentration. The datasets shown in the graphs represent the averages of the results of four (fluvoxamine) or two (epalrestat) individual experiments.

To probe the role of CYP4A11 and CYP1A2 in MONACRA metabolism by HLM, we employed inhibitory analysis with the use of epalrestat as a specific inhibitor of CYP4A11 [22] and fluvoxamine as a CYP1A2 inhibitor [37]. We explored the effect of the inhibitors on the activity of recombinant CYP1A2 and CYP4A11, as well as the activity of six different preparations of HLM (preparation X347 was not available at the time of the study). The results of these experiments are shown in Figure 12.

As seen from Figure 12A, the effect of epalrestat on CYP1A2 is complex but modest. Surprisingly, it shows a slight activation of CYP1A2 at low concentrations, which changes to a slight inhibition as its concentration increases. In contrast, consistent with the literature data [22], epalrestat exhibits complete inhibition of CYP4A11 characterized by *IC*_50_ of 1.3 µM. In all studied HLM samples, epalrestat inhibited MONACRA with an amplitude of inhibition of 97.2±2.5 and *IC*_50_ of 3.0±1.4 µM.

The parameters of inhibition in six studied HLM preparations, along with the fractions of three Michaelis–Menten components of their SSPs, are shown in Table 2. As seen from this table, the amplitude of inhibition was close to 100% in all preparations, regardless of the distribution of the amplitudes of high-, middle-, and low-affinity components (*F*_1_, *F*_2_, and *F*_3_ in Table 2). This observation suggests that all three Michaelis–Menten components reflect the activity of CYP4A11. In agreement with this conclusion, the value of *IC*_50_ exhibited by six HLM samples reveals a significant correlation (*R*=0.869) with the fraction of the high-affinity phase. This relationship is quite understandable since the *IC*_50_ value is determined by the values of *K*_D_ of the enzyme–substrate complex, *K*_i_, and substrate concentration. An increase in the enzyme’s affinity to the substrate (or an increase in the contribution of the high-affinity component, as in our case) would lead to an increase in the *IC*_50_ value. We can hypothesize that the presence of the high- and low-affinity components of MONACRA SSPs is due to the presence of the fractions of the enzyme, where its affinity to the substrate is changed, potentially due to interactions with other P450 species or other microsomal proteins. In this regard, our observation of the correlation between the amplitude of the high-affinity component of SSPs and the *b*_5_ abundance is noteworthy. It may indicate the impact of the interactions of *b*_5_ with CYP4A11 on its affinity to the substrate.

**Table 2:** Distribution of the amplitudes of three Michaelis–Menten components of 3-(6-methoxynaphthalen-2-yl)acrylic acid (MONACRA) demethylation and parameters of its inhibition by epalrestat in six pooled human liver microsome (HLM) preparations.^1^

HLM sample	*F*_1_, %	*F*_2_, %	*F*_3_, %	*IC*_50_	Inhibition, %
CDN	0.1	68.7	31.2	1.48±0.21	99.7±0.6
DNJ	0.5	91.6	7.9	2.02±1.70	93.0±2.3
EGW	2.5	97.5	0.0	2.15±0.52	99.8±0.4
WGP	0.0	34.4	65.6	2.14±0.94	97.1±5.7
X096	9.8	0.0	90.2	5.36±0.13	93.9±0.7
X263	21.7	21.5	56.8	5.09±1.04	100.0

1 The values *F*_1_, *F*_2_, and *F*_3_ represent the fractional contribution of the high-, middle-, and low-affinity phases to the SSPs of MONACRA metabolism by HLM samples. The values of *IC*_50_ and the inhibition amplitude are the averages of the results of the two experiments. The ± values are the confidence intervals calculated for *P*=0.05.

The effect of fluvoxamine on MONACRA metabolism is illustrated in Figure 12B. It exhibits a profound inhibition of CYP1A2 in Supersomes with an amplitude of 95% and *IC*_50_ of 0.56 µM. In contrast, it exerts a pronounced activation on MONACRA metabolism by CYP4A11 Supersomes. Unexpectedly, its effect on MONACRA metabolism in HLM is complex and varies between the probed preparations. The preparations EGW and X263 exhibit activation similar to that observed in CYP4A11 Supersomes. CDN, WGP, and X096 demonstrate a combination of an inhibitory effect at lower concentrations with an activation that manifests itself as the concentration increases. In the case of DNJ, the activation is not pronounced, and the inhibition is characterized by *IC*_50_ of 14 µM and amplitude of 69%. The reasons for these differences remain unclear. However, they show that the effect of fluvoxamine is not due to the direct involvement of CYP1A2 in MONACRA metabolism. Instead, it reveals its impact on protein–protein interactions within the P450 ensemble, whose composition varies significantly between the HLM samples. Overall, our inhibitory analysis implicates CYP4A11 as the sole metabolizer of MONACRA in HLM and does not reveal any significant role for CYP1A2 in MONACRA demethylation.

## Conclusions

This study introduces MONACRA as a specific fluorogenic substrate of CYP4A11, the major human enzyme responsible for 20-HETE formation. Probing the activity of 16 microsomal P450 enzymes with this substrate, we demonstrated that besides CYP4A11, the only enzyme exhibiting appreciable activity in MONACRA demethylation is CYP1A2. However, our inhibitory analysis did not reveal any involvement of CYP1A2 in MONACRA metabolism in HLM. According to our results, CYP4A11 is responsible for >93% of MONACRA demethylation in all studied HLM samples. Therefore, MONACRA can be used as a specific fluorogenic substrate of CYP4A11. The strong fluorescence of HONACRA, the MONACRA demethylation product, is well resolved from the substrate’s fluorescence at alkaline pH. This feature allowed us to develop a reliable and sensitive automated fluorimetric assay applicable for high-throughput screening of CYP4A11 activity in large series of tissue specimens. Such screening will be instrumental in investigating the correlations between the composition of the P450 ensemble and the activity of CYP4A11, the key enzyme in synthesizing 20-HETE in the human liver. Combining this approach with proteomic characterization of the individual samples in HLM collections will allow exploring the impact of various environmental and temporal factors affecting P450 expression on 20-HETE-intermediated signaling. The studies of the effect of chronic alcohol exposure on CYP4A11 activity using this approach are now ongoing.

## Materials and methods

### Chemicals

3-(6-Methoxynaphthalen-2-yl)acrylic acid was a product of BOC Sciences (Shirley, NY, U.S.A.). Fluvoxamine and epalrestat were purchased from APExBio (Houston, TX, U.S.A.). Acetoacetanilide was procured from the Tokyo Chemical Industry (Tokyo, Japan). Coumarin 152 was a product of Acros Organics, now a part of Thermo Fisher Scientific (Waltham, MA, U.S.A.). Glucose-6-phosphate dehydrogenase from *Leuconostoc mesenteroides*, NADP, and glucose-6-phosphate were the products of MilliporeSigma (Burlington, MA, U.S.A.). LC-MS grade acetonitrile, water, and formic acid were purchased from Fisher Scientific (Fair Lawn, NJ). All other reagents were of ACS grade and used without additional purification.

### Pooled human liver microsomes and monkey liver microsomes

This study used preparations of pooled HLMs from 50, 150, or 200 donors (mixed gender). The preparations CDN, DNJ, and WGP were INVITROCYP 150-Donor Human Liver Microsomes obtained from BioIVT Corporation (Westbury, NY). Lot EGW is INVITROCYP M-Class 50-Donor HLM procured from the same company. Lots 1,910,096 and 1,210,347 were XTreme 200 Mixed Gender HLM (200 donors) purchased from XenoTech LLC (Kansas City, KS), currently a part of BioIVT. Lot 2,110,263 corresponds to mixed gender HLM from 50 donors from the same company. These lots are referred to herein as X096, X347, and X263, respectively. The supplier of the HLM preparations used in our studies, BioIVT Corporation, has declared to adhere to the regulations of the Department of Health and Human Services for the protection of human subjects (45 CFR §46.116 and §46.117) and Good Clinical Practice, (ICH E6) in obtaining the samples of human tissues used for producing the preparations of human subcellular fractions available from these companies.

Liver microsomes from Cynomologus monkey and Sprague-Dawley rats were procured from GIBCO, a part of Thermo Fisher Scientific (Waltham, MA, U.S.A.).

### Microsomes containing recombinant human cytochromes P450

Most of the preparations of insect cell microsomal containing baculovirus-expressed individual P450 enzymes (Supersomes^TM^) were the products of Gentest (Huntsville, AL), now a part of Discovery Life Sciences (Woburn, MA). In the present study, we used the preparations containing CYP2B6 (lot 31487), CYP2C8 (lot 81760), CYP2C9 (lot 75854), CYP2C19 (lot 73445), CYP2D6 (lot 2309291), CYP2E1 (lot 23012), CYP3A4 (lot 81745), CYP3A5 (lot 89573), CYP2J2 (lot 2215966), CYP4F2 (lot 23112130), CYP4F3A (lot 3), CYP4F3B (lot 2), CYP4F12 (lot 29692), and CYP4A11 (lot 2402280). The preparation of insect cell microsomes containing human CYP2A6 (lot 2520331) along with human CPR (Baculosomes®) was procured from Thermo Fisher Scientific (Waltham, MA, U.S.A.). All those preparations, except for the CYP1A2 and CYP4A11 Supersomes, also contained human cytochrome *b*_5_ co-expressed.

### Characterization of the content of protein, NADPH-cytochrome P450 reductase, and cytochromes P450 and b_5_ in HLM

Protein concentrations in microsomal suspensions were determined by the bicinchoninic acid assay [38]. The concentration of CPR in microsomal membranes was determined based on the rate of NADPH-dependent reduction in cytochrome *c* at 25°C, and the effective molar concentration of CPR was estimated using the turnover number of 3750 min^−1^ [39]. The total concentration of cytochromes P450 in HLMs was determined with a variant of the ‘oxidized CO versus reduced CO difference spectrum’ method described earlier [39].

### Fluorescence spectroscopy

Spectra of emission and excitation of fluorescence were acquired with a Cary Eclipse fluorometer (Agilent Technologies, Santa Clara, CA, U.S.A.) equipped with either a thermostatted cell holder and motorless magnetic stirrer (Variomag-USA, Port Orange, FL, U.S.A.) or a fluorescence plate reader accessory.

### High-throughput fluorimetric assays of MONACRA metabolism

The process of developing this procedure is outlined in ‘Elaborating the fluorimetric assay of MONACRA demethylation’ under Results. The final protocol of the high-throughput assay with the use of an OT-2 liquid handling robot (Opentrons Inc., Brooklyn, NY) and Cary Eclipse fluorometer equipped with a plate reader accessory (Agilent Technologies, Santa Clara, CA, U.S.A.) is described below.

The MONACRA metabolism experiments were conducted with 12 MONACRA stock solutions in the Incubation Buffer (0.1 M Hepes buffer pH 7.4 containing 60 mM KCl) with concentrations ranging from 0 to 3.3 mM prepared with serial dilution. A 0.3 M stock solution of MONACRA in DMSO was diluted by the Incubation Buffer to prepare the most concentrated (3.3 mM) solution in the series. This series of stock solutions provided the MONACRA concentrations in the incubation mixture decreasing from 825 to 0 µM with a dilution factor of 1.73333 (≈√3). The procedure of preparing the multiwell plates for activity assays was similar to that described earlier for FA-based assays [34]. The prepared plate was placed into a heater-shaker module with a PCR-plate adapter in location 1 of the OT-2 deck. Suspensions of microsomes were diluted to the desired concentration (0.02–0.1 µM P450 for recombinant enzymes and 0.3–1 mg/ml for HLM samples) and placed into all eight wells of the first row of a PCR plate (96-well semi-skirted PCR plate, BrandTech Scientific Inc., Essex, CT), 255 µl per well. The PCR plate was inserted into a pre-cooled PCR-plate cooling block (Eppendorf, Hamburg, Germany) and placed at position 5 of the OT-2 deck. An Azzota deep-well plate placed in location 6 contained 0.4 ml of 0.3 M solution trichloroacetic acid as a quenching reagent. The second row of the same plate contained 0.6 ml of 2.4 molar Na-glycine buffer, pH 10.4. The protocol of the OT-2 run of the activity assay starts with pre-incubation of the reaction plate in the heater-shaker module set at 1200 rpm shaking and 37°C. This setting of the heater resulted in the temperature of 29.5–31°C in the incubation mixture stabilized after 8 min of pre-incubation. Subsequently, the robot starts the reaction by dispensing 20 µl of microsomal suspension per well with an 8-channel 300 µl pipet. The addition of microsomes to each row was followed by 20-sec shaking. After adding microsomes to the last row, the shaking continues until the desired incubation time (20 min). The reaction was then stopped by adding the quenching solution in the volume of 32 µl per well with an 8-channel 300 µl pipette. The addition of the reagent to each row was followed by shaking for 20 sec. The protocol ends with adding the alkaline Na-glycine buffer in the volume of 48 µl per well. The OT-2-compatible protocol file for this procedure is available from the authors on request.

The incubation plate treated as described above was then centrifuged at 3700 rpm in a Beckman Allegra 6R centrifuge with a GH-3.8 swing-out rotor with multiwell-plate adapters. After 15 min of centrifugation, the plate was scanned with a Cary Eclipse fluorometer with a well-plate accessory. We acquired the spectra of fluorescence emission in the 416–650 nm region with excitation at 375 nm and the slits set at 20-nm bandwidth.

### Assays of MONACRA oxidative demethylation with determination of formed formaldehyde

MONACRA demethylation was monitored by quantifying the amount of formed formaldehyde using a high-throughput fluorimetric assay based on the Hantzsch reaction with acetoacetanilide. The plates were prepared as described above. The assays were executed as described earlier [11,34], except for monitoring the formation of DPDL by the spectra of its emission with excitation at 395 nm instead of the spectra of excitation, as it was described earlier. This modification of the assay procedure was done because of an ample overlap of the spectra of excitation of MONACRA and HONACRA with that of DPDL.

### Assays of MONACRA metabolism with LC-MS technique

Quantification of MONACRA, HONACRA, and DPDL (the product of Hantzsch reaction of acetanilide with FA) was performed using targeted multiple reaction monitoring (MRM)-based assay on microflow M-Class UPLC hyphenated with Xevo-TQ-XS mass spectrometer (Waters, Milford, MA). Acquity UPLC HSS T3 column (100  Å, 1.8  μm, 1 mm × 100 mm) connected with a Vanguard™ pre-column (2.1 mm × 5 mm, 1.8 μm particle size) was used for metabolite elution. The LC parameters were set at a 50  μl/min flow rate with a 1  μl injection volume and a column temperature of 40°C. Mobile phases A (water with 0.1% formic acid) and B (acetonitrile with 0.1% formic acid) were used with a gradient elution program. The initial gradient of 30% B was held for 0.8 min, followed by a linear increase to 70% B (0.8–2.5 min), 70–90% B (2.5–4.3 min), 90% (4.3–5.3 min), and 30% B (5.3–7.5 min). For MS data acquisition, MRM transitions were as follows: MONACRA (m/z 229.1→211.1 at CE 20 eV, m/z 229.1→183.1 at CE 25 eV, CV 30), HONACRA (m/z 215.1→197.1 and m/z 215.1→169.1, CE 25 eV, CV 30), DPDL (m/z 348.2→201.1, 148.1, 118.1, 110.1, CE 20 eV, CV 30), and Midazolam (m/z 326.1→309.1, 291.1, 244.0, CE 20 eV, CV 35). Elution times for compounds were 5.3 min for MONACRA, 4.5 min for HONACRA, 4.3 min for DPDL, and 4.1 min for midazolam. Midazolam was used as an internal standard. Peak integration and quantification were performed using Skyline 24.1 (University of Washington). Quantification was accomplished using a linear regression fit to an external calibration curve prepared in tandem with samples. Calibration curves of MONACRA (7.8–4000 ng/ml) prepared in a mixture of 0.1% formic acid in water and acetonitrile (50:50 v/v) were used for calculating metabolite concentrations in HLM incubations. Midazolam (0.1 µM) was spiked in each calibration curve preparation. MONACRA incubated with enzymes for quantification of substrate depletion and metabolite formation was diluted 100-fold before LC-MS analysis.

### Kinetic assays of MONACRA metabolism

Assays of the time course of MONACRA depletion and the formation of the products of its metabolism were carried out in a way similar to that described above for high-throughput fluorimetric assays of MONACRA metabolism but without the use of an OT-2 robot. The multiwell plates containing 70 µl of incubation buffer, NADPH-generating system, and 171.4 µM MONACRA per well were placed into an Incu-Mixer MP2 heated microplate shaker (Benchmark Scientific, Sayreville, NJ, U.S.A.) and incubated to reach the temperature of 36°C at a shaking speed of 600 rpm. The reaction was started sequentially from the first to the 12th rows of the plate with a 2-min time interval between rows by adding 10 µl of microsomal suspension per well. After adding microsomes to the penultimate (11th) row of the plate, at the 22nd minute of incubation, the reaction was stopped by the addition of 32 µl of AAA reagent (in the case of formaldehyde assays) or 0.3 M TCA (in the case of HONACRA fluorescence assays) to all 12 wells of the plate column with a multichannel pipette. It was followed by adding 10 µl of microsomal suspension to the 12th row of the plate. The wells were then supplemented with 48 µl of the ammonium acetate solution (in the FA-AAA assay case) or alkaline Na-glycine buffer (in the case of fluorimetric HONACRA assays) and analyzed as described above.

### Enzyme inhibition experiments

Inhibition of MONACRA metabolism by epalrestat and fluvoxamine was studied using the high-throughput assay described above. The substrate concentration was kept constant at 150 µM. Each column of the well plate comprised eight wells with different concentrations of inhibitor complemented with two wells with no inhibitor added (100% activity samples) and two wells with no inhibitor and no NADPH added (‘blank’ samples). The concentration of inhibitors in the series of eight samples with added inhibitor decreased from 63 µM (epalrestat) or 32 µM (fluvoxamine) with a dilution factor of 1.7333 (≈√3). The fractional inhibition at each inhibitor concentration was determined from the ratio of the reaction rate in the presence of an inhibitor to the rate observed at no inhibitor added. The resulting dependence of the fractional inhibition on the inhibitor concentration was fitted to a four-parameter logistic equation (Hill equation) [40]:


(1) $$F_{I}=\frac{v_{0}-v_{i}}{v_{0}}=A_{max}\frac{[I{]}^{N}}{IC_{50}+[I{]}^{N}}$$

In this equation, *F*_I_ and $v_{i}$ are the fractional inhibition and the reaction velocity observed at inhibitor concentration [I], $v_{0}$ is the reaction rate at no inhibitor added, and *A*_max_ is the maximal amplitude of inhibition. *IC*_50_ is the concentration of inhibitor at which the inhibition reaches 50% of its maximal amplitude (*A*_max_), and *N* is the Hill coefficient.

### Global kinetic analysis of substrate saturation profiles

The datasets for global kinetic analysis were assembled as the sets of averages of 2–4 SSPs obtained with each HLM preparation. The combined dataset for all seven HLM preparations was subjected to PCA. The results of PCA were used to find a set of three Michaelis–Menten equations whose linear combinations best approximate each of the individual SSPs as described earlier [11]. All manipulations with the dataset, PCA, and regression analysis were performed using our SpectraLab software [41,42], available for download at http://cyp3a4.chem.wsu.edu/spectralab.html.

### Analysis of substrate saturation profiles for individual recombinant P450 enzymes

SSPs for individual recombinant P450 enzymes were approximated with either the Michaelis–Menten equation or, in the case of CYP1A2, with the equation of substrate inhibition ([43], eqn 5.9):


(2) $$v=\frac{v_{max}\cdot [S]}{K_{M}+[S]+[S{]}^{2}/K_{si}}$$

where v and $v_{max}$ are the reaction rate at substrate concentration [*S*] and the maximal reaction rate, respectively, *K_M_* is the Michaelis constant, and *K_si_* is the substrate inhibition constant. The experimental datasets were fitted to these equations using a combination of Nelder–Mead and Marquardt non-linear regression algorithms implemented in the SpectraLab software.

### Molecular modeling and docking

The model of human CYP4A11 structure was built using AlphaFold [32]. The heme moiety was inserted by superimposing the AlphaFold-generated structure of the protein chain with the crystal structure of CYP4B1 (PDB entry 5T6Q) using the Chimera 1.18 package from the Computer Graphics Laboratory, University of California, San Francisco (http://www.cgl.ucsf.edu/chimera/, [33]). In both proteins, the porphyrin ring is bound to the protein by a covalent bond between its 5-methyl group with the carboxylic acid side chain of a conserved glutamate residue in the helix I (Glu_310_ in CYP4B1 and Glu_321_ in CYP4A11) [44,45]. This bond was added by editing the PDB file obtained after inserting the coordinates of CYP4B1 heme moiety into the AlphaFold-generated CYP4A11 structure. Analysis of the resulting structure with Chimera 1.18 did not reveal any critical clashes in the structure produced by the heme insertion. Docking of the ARA and MONACRA molecules into this structure was performed using Autodock Vina [31]. The docking cage of 20×20×20 Å was centered around the coordinates of the β-oxygen atom of the Glu_321_ side chain.

## Supplementary material

Online supplementary material

## Data Availability

The data are contained within the article. The raw datasets used to generate the reported results are available from the authors upon reasonable request.

## Abbreviations

ARA: arachidonic acid
C152: Coumarin 152
CPR: NADPH-cytochrome P450 reductase
DDPL: 3,5-di-N-phenylacetyl-1,4-dihydrolutidine
FA: formaldehyde
FA-AAA: formed formaldehyde with acetoacetanylide
20-HETE: 20-hydroxyeicosatetraenoic acid
HLM: human liver microsome
HONACRA: hydroxy-naphthyl acrylic acid
MLM: monkey liver microsomes
MONACRA: 3-(6-methoxynaphthalen-2-yl)acrylic acid
MRM: multiple reaction monitoring
SSP: substrate saturation profile
