# Supplementary material for: A new naphthalene-based fluorogenic substrate for cytochrome P450 4A11
Source: Biochem J. 2025 Jun 17;482(12):839–57. doi: 10.1042/BCJ20253130 (PMC12191924; doi:10.1042/BCJ20253130)
Supplement: Online supplementary material [file bcj-482-12-BCJ20253130-supp1.pdf]

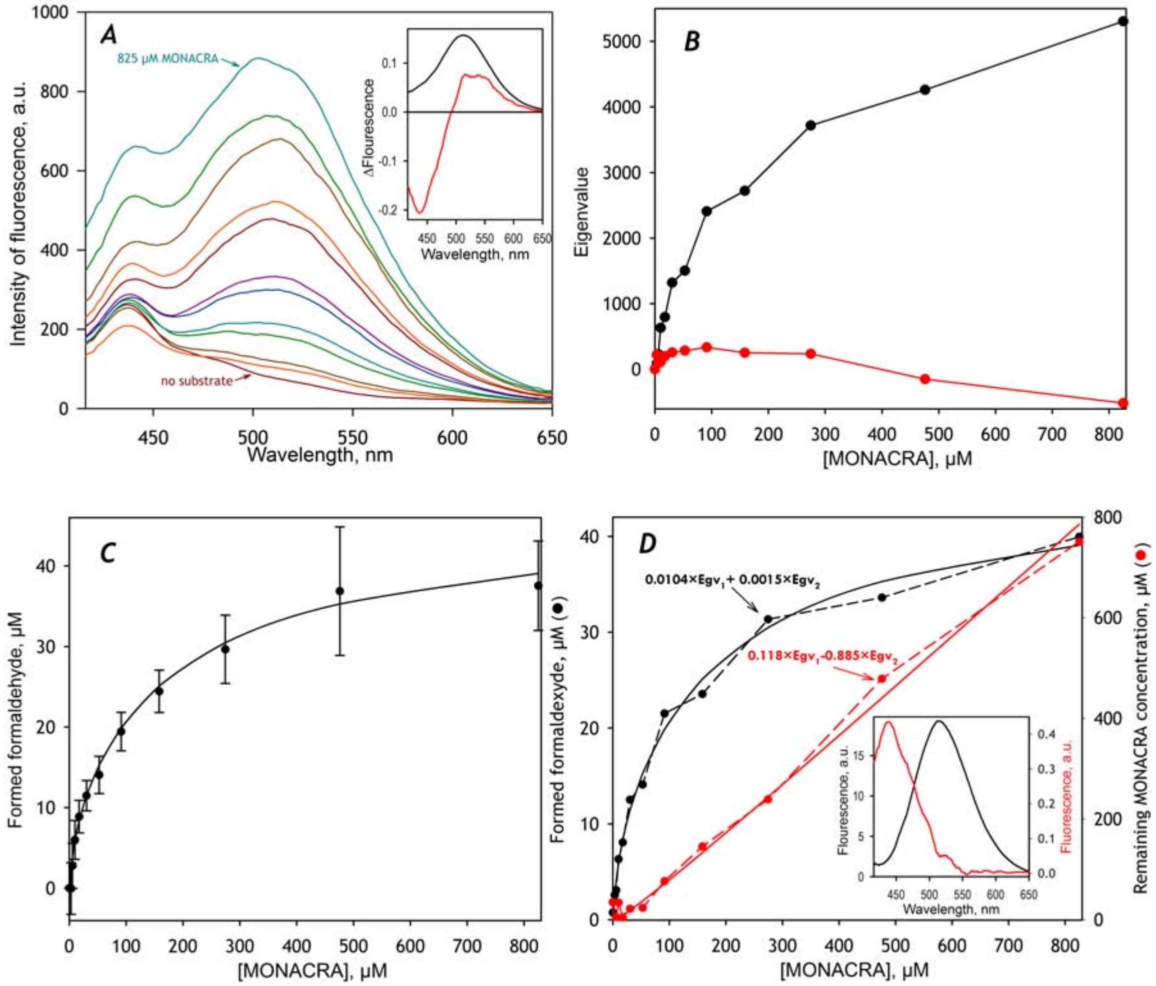

<u>Figure S1.</u> Derivation of the spectrum of HONACRA fluorescence from the results of fluorimetric activity assay with rat liver microsomes (RLM, 1.96 mg/ml) and MONACRA. Panel **A** shows a series of emission spectra (excitation at 375 nm, excitation and emission slits of 10 and 20 nm, respectively, and the PMT voltage of 700 V) obtained after 20 min RLM incubation at 30 °C with NADPH-generating system and MONACRA at concentrations from 825 to 3.4 μM with a dilution factor of 1.7333. The inset shows the spectra of the first (black) and second (red) principal components obtained by PCA applied to this dataset. The respective vectors of eigenvalues are shown in Panel **B**. Panel **C** shows the results of determining formed formaldehyde in a parallel experiment in the same conditions. The data points represent the averages of two individual assays, and the error bars show the respective standard deviations. The solid line shows the fitting of this dataset with a combination of two Michaelis-Menten equations (K_{M1}=12 μM, K_{M2}=192 μM). The dependencies of the formaldehyde concentrations (black) and remaining MONACRA (red) calculated from this fitting are shown in Panel **D** with solid lines. The filled circles and dashed lines show the approximations of these dependencies with the combinations of the first two eigenvectors (Egv_1 and Egv_2, respectively). This approximation results in the following matrix of multiplication factors: $\begin{vmatrix} 1.04\cdot10^{-2} & 1.47\cdot10^{-3} \\ 0.118 & -0.885 \end{vmatrix}$. By inverting and transposing it, we obtain the following: $\begin{vmatrix} 93.5 & 18.7 \\ 0.156 & -1.1 \end{vmatrix}$. Combining the first and the second principal components (Panel A, inset) with the coefficients from the first row of the inverted and transposed matrix gives us the fluorescence spectrum of 1μM HONACRA (inset, black line). Using the coefficients from the second row, we obtain the 1 μM MONACRA spectrum (inset, red line).

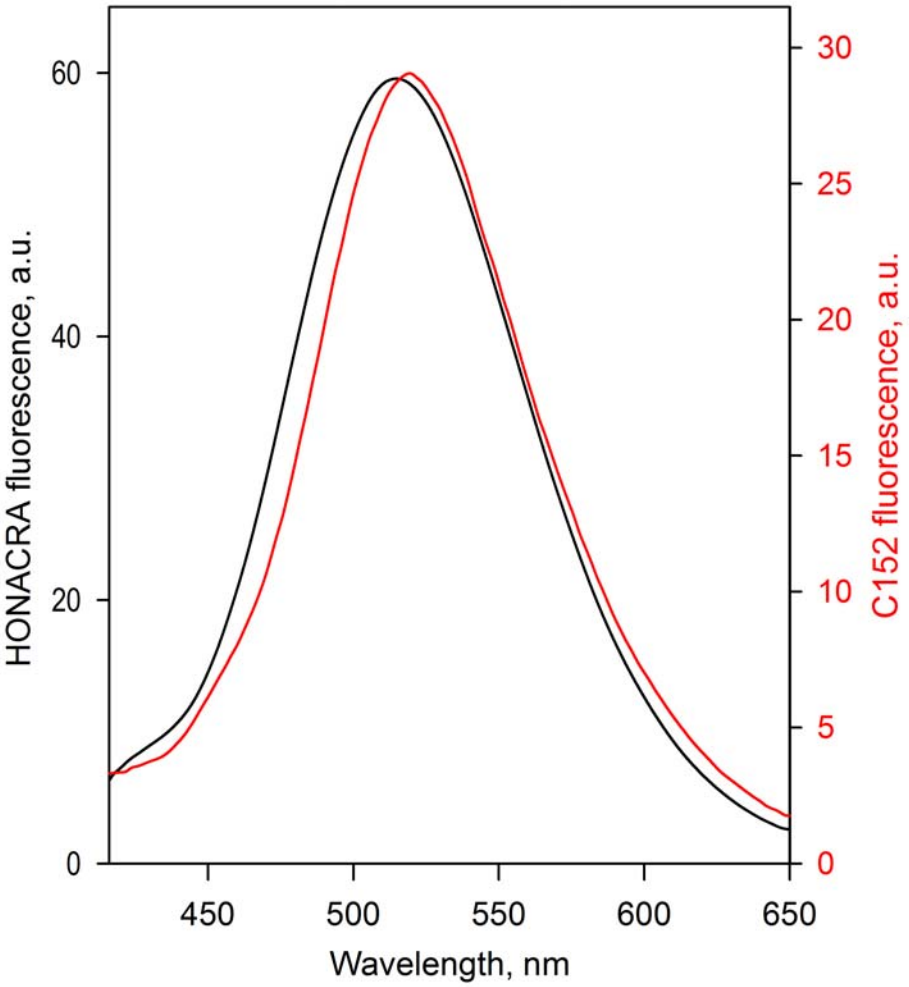

<u>Figure S2</u>. Emission spectra of 1 μM HONACRA (black) and 1 μM Coumarin 152 (red) in our assay media (pH 10.4) taken with excitation at 375 nm (excitation and emission slits of 10 and 20 nm, respectively, PMT voltage of 800 V).
